# Cognitive-Affective Change Mechanisms in Personalized Normative Feedback via the Articulated Thoughts in Simulated Situations Paradigm

**DOI:** 10.3390/ijerph17030690

**Published:** 2020-01-21

**Authors:** Justin F. Hummer, Melissa R. Hatch, Gerald C. Davison

**Affiliations:** 1 Behavioral & Policy Sciences, RAND Corporation, 1776 Main Street, Santa Monica, CA 90407, USA; 2 Center for Alcohol and Addiction Studies, Brown University School of Public Health, Providence, RI 02903, USA; melissa_hatch@brown.edu; 3 Laboratory for Cognitive Studies in Clinical Psychology, Department of Psychology, University of Southern California, Los Angeles, CA 90089, USA; gdaviso@usc.edu

**Keywords:** alcohol use, personalized normative feedback, PNF, adjudicated students, college students, Articulated Thoughts in Simulated Situations, ATSS, mechanisms of change

## Abstract

The research explored explanatory mechanisms of change for a personalized normative feedback (PNF) intervention, through an adapted application of the Articulated Thoughts in Simulated Situation (ATSS) cognitive think-aloud paradigm. A sample of 70 (51% female) U.S. adjudicated students were randomly assigned to one of three conditions: a PNF-ATSS condition, a PNF-Only condition (without ATSS), and an active Control+ATSS condition which received psychoeducation about alcohol use. Students in both the PNF-Only and PNF-ATSS conditions reported significant reductions in their misperceived peer drinking norms and alcohol-related consequences at the 30-day follow-up, relative to students in the control condition. Participants in the PNF-ATSS condition drank significantly fewer drinks per week at follow-up than participants in the PNF-Only condition, but not less than participants in the control condition. Significant indirect effects were found for the ATSS codes of participants’ neutrality and believability toward PNF content. This study presents a proof of concept for an adapted ATSS think-aloud methodology as a clinical science intervention tool to specify the cognitive-affective processes of change linked to complex intervention for particular problems, persons, and contexts.

## 1. Introduction

Problematic drinking by college students is a public health concern. National data in the United States (U.S.) indicate most young adults drink alcohol, with about one-third of young people between 18 and 25 engaging in heavy drinking (five or more drinks in a row in the previous two weeks) and about one in 10 young adults reporting consumption of 10 or more drinks in a row during just the previous two weeks [1]. The consequences of heavy drinking by young adults are well documented and include academic problems, physical injuries and fights, risky sexual behavior and sexual assaults, memory blackouts and passing out, sustained cognitive deficits, alcohol poisoning, and even death [2,3,4]. Despite significant resources and efforts dedicated to reducing alcohol harm, problematic alcohol use in U.S. colleges remains widespread. 

### 1.1. Adjudicated Students

Adjudicated (also called mandated) students are those who receive sanctions for violating the alcohol use policy at their college or university. Most if not all U.S. colleges have some policy prohibiting drinking in their under-age student body, as well as consequences for its violation that may include monetary fines, informing parents, and mandated activities [5], but recidivism rates and risk levels remain high [6]. In fact, there have been consistent increases in the number of alcohol-related arrests, the number of students receiving alcohol citations, and the proportion of students mandated to participate in a post-citation intervention on U.S. college campuses [7,8,9], highlighting the need to focus on the development, effectiveness, and sustainability of adjudicated-student intervention programs. 

Interventions for adjudicated students have demonstrated improved outcomes relative to control conditions and have been shown to be effective short-term risk reduction strategies [10]. Due to the low number of no-treatment or waitlist control studies conducted with adjudicated students, a recent review was able to determine only that any intervention produces better outcomes than no intervention [10]. This highlights the need for a more systematic analysis of the efficacy of intervention components. 

### 1.2. Social Norms and Personalized Normative Feedback 

Social influences and student perceptions of the prevalence of peer drinking factor heavily into personal decisions about when and how much to drink [11,12,13,14]. However, the vast majority of college students from many different countries overestimate how much and how frequently other students drink [15,16,17,18,19,20,21]. Personalized normative feedback (PNF) is one-time individually delivered information designed to correct normative misperceptions. Based on data demonstrating the strong association between perceived norms and alcohol use in college populations, correction of normative misperceptions using PNF is a prominent focus of many college drinking intervention studies [22,23,24,25,26]. When provided to heavier drinking participants, PNF is designed to highlight two pieces of information regarding normative beliefs known to influence drinking behavior, namely: (1) other students drink less than the participant drinks (social comparison information provided the individual’s alcohol use is heavier than the prevailing norm), and (2) other students drink less than the participant thinks they drink (normative misperception correction). The idea is to correct normative misperceptions and motivate behavior change by showing discrepancies between students’ perceptions and others’ self-reported behaviors [27]. 

Stand-alone computerized and web-based PNF interventions in the U.S. have been found to reduce alcohol use in randomized clinical trials, with effect sizes typically in the small range [28]. Outside of the U.S., PNF interventions have had mixed success. For example, one large RCT in the United Kingdom (UK) observed that web-based PNF effects on reductions in alcohol use were sustained 19 weeks post-intervention [29], while another large-scale RCT in the UK found no evidence of PNF effects on alcohol outcomes [30]. In light of the mixed international findings regarding the efficacy of computerized and web-based PNF as an intervention strategy for reducing college student drinking, and the growth potential to achieve stronger and longer-term outcomes, continued research seeking to better understand, culturally adapt and enhance PNF is warranted. The present study, in part, represents an effort to do so.

### 1.3. Exploring Change Mechanisms of PNF Intervention Efficacy

Exploring mechanisms of change, the causal links between treatment and outcomes, may help clarify the processes that are critical to PNF and how it operates (what leads to change and why). To date, several studies comparing PNF to appropriate control groups have shown that changes in perceived norms are associated with changes in drinking [31,32,33]. Although other mediators have been examined, none have been supported, and there is limited understanding as to why [10].

In two studies targeting adjudicated students, multicomponent web-based PNF was associated with reductions in drinking outcomes compared to active comparison groups and the effects of the intervention were partially accounted for by the changes in estimates of peer drinking [7,34]. These studies provide good support for the theoretically derived mechanism of change in PNF interventions (i.e., reduction in normative misperceptions), yet they are still limited by the focus on only one change agent, which did not capture cognitive elements of participants’ reactions. In addition, the studies were performed in the context of web-based multicomponent interventions that included PNF in addition to other alcohol intervention information (e.g., approximate financial cost of drinking, health effects), rather than the traditional stand-alone, mono-component PNF, making it difficult to ascertain which components were most responsible for reductions in drinking. They also assessed the mediator and outcomes concurrently, which deviates from the recommended guidelines for establishing mediation. As with other PNF interventions, the studies found no effects of the intervention on alcohol consequences. 

### 1.4. A Novel Methodological Approach: Articulated Thoughts in Simulated Situations (ATSS)

The Articulated Thoughts in Simulated Situations (ATSS) experimental paradigm is a laboratory-based think-aloud approach designed to capture complex cognition and emotion through open-ended responding to specific stimuli presented by the experimenter [35]. Traditionally, ATSS involves having people imagine that they are participants in a situation/vignette and to verbalize their thoughts and feelings during pauses in the story. The scenario is typically presented in short segments or “doses”, after each of which the participant articulates his thoughts and feelings. Because the ATSS paradigm is concerned with a person’s immediate experience, responding does not rely on long-term memory and therefore avoids the problems of retrospective self-reporting. The articulated thoughts themselves are recorded, transcribed, and later content-analyzed. Since the introduction of the paradigm, over 100 published studies attest to the validity of ATSS in accessing complex cognition across a wide array of domains [36,37].

The ATSS paradigm provides a cognitive-affective and phenomenological framework that may be useful in capturing participants’ impressions of the PNF intervention material and in so doing, potentially uncover additional mechanisms of change. Rather than its traditional use within the context of a “simulated situation”, the ATSS approach was modified to consider the participants’ immediate reactions to each specific component (i.e., dose) encompassing the complete PNF package as it is displayed to the participant. The application of the ATSS to studying how participants actively construe information during an intervention is a new direction for the paradigm, with potentially fruitful implications for broader evaluations of public health interventions. 

As noted above, this paradigm assesses thoughts as they occur (rather than relying on post factum reports) and does not limit response format while simultaneously allowing for experimenter control. Because it allows for the participant to respond with any and all thoughts that have occurred to her, ATSS seems to have particularly high utility when “little is known of the cognitive terrain of interest” [36] (p. 955). ATSS will allow us to display the intervention content for a participant while providing access to our participants’ real-time cognitions in response to the intervention. In this way, ATSS has the potential to provide insight into the in vivo cognitive processes that are occurring during PNF and that may be instrumental in behavior change. We expected that a detailed examination of the cognitions and emotions experienced during PNF exposure would yield insight into the mechanisms that account for changes in outcome data.

#### Cognitive-Affective Change Mechanisms

Although it is challenging to offer a priori hypotheses for the emergence of specific cognitive-affective reactions that may account for intervention efficacy, due to the dearth of research on the topic, it is useful to provide what we believe might be some examples. First, if participants are skeptical with respect to the source of the normative feedback, and thus its validity, the effectiveness of social norms interventions may be diminished [38,39,40]. It is generally understood by social norms practitioners that students experience varying degrees of skepticism related to the normative information included in these interventions [14]. Discounting the credibility of the normative feedback will allow heavy-drinking students to continue their level of drinking without experiencing the sense of conflict elicited by the knowledge that they are deviating from the prevailing norm, the presumed mechanism of change within PNF interventions. 

It is likely that some students may discount PNF data because they may not believe that the surveyed students represent a true sampling of students or of their drinking behavior, or they may believe that the researchers manipulated the data by providing incorrect information in an attempt to persuade the student to change. Despite our intention to adequately highlight the reliability and source of the data used in the feedback, we expected some variability in the extent to which students find the information credible/believable and anticipated that this coding theme will emerge in the ATSS data. 

The current study further assessed for the presence of ATSS codes that capture expressions of surprise/shock, neutrality, and self-reflection in response to the information presented. It was expected that each of these codes, if present, would play a role in changing perceived norms and alcohol use behaviors. The success of this study and its ability to evaluate novel cognitive-affective variables related to mechanisms of change are strengthened by its implementation in a laboratory setting, the real-time collection of cognitions and emotions, and the unconstrained response format. As already noted, process research may provide a better understanding of the key mechanisms of change. This will be of importance both theoretically and in a broader applied realm. It may help improve the effectiveness of PNF and concurrently evaluate the ability of an innovative methodological approach to provide important theoretical and practical contributions to the larger social and clinical psychological literature on the underpinnings of PNF for health behavior change.

### 1.5. ATSS and Depth of Processing

One potential concern with using the ATSS paradigm as a purely cognitive assessment device within an active intervention is that it may impact an individual’s processing of the information. That is, having participants pause periodically during the PNF to express what is on their mind may either distract the participants or confer additive therapeutic effects beyond that which arises purely from the content presented. When one receives information, it can be processed with varying levels of cognitive engagement. Depth of processing refers to the extent to which individuals expend cognitive effort and think more carefully about the information provided in persuasive communication. Because the ATSS paradigm has heretofore not been utilized in the fashion proposed for this study, it remained unknown if the inclusion of ATSS would enhance the effectiveness of conditions by deepening an individual’s processing of the content presented, beyond the effects of content delivered sans ATSS. In other words, ATSS as an assessment methodology may have change properties. To account for potential ATSS reactivity effects, we included two intervention conditions; one condition included viewing standard PNF without the think-aloud ATSS portion (PNF-Only), and the other condition included PNF with ATSS (PNF-ATSS).

### 1.6. Objectives of the Current Study 

Although exploratory in nature, we expected that a more qualitatively detailed examination of the cognitions and emotions experienced during PNF exposure would yield insight into other mechanisms that account for any positive outcome data. The ATSS experimental paradigm was adapted from its more traditional applications in hypothetical and simulated scenarios to a laboratory-based, process-oriented assessment of a PNF intervention. Interventions were delivered individually on computer in a lab setting and used pre/post assessments to monitor changes in study outcomes. These measures address limitations in previous research while allowing for a high degree of experimenter control and increased the potential to identify mechanisms of change within the intervention. Both conditions were compared to an active comparison group that received information about college students and alcohol use derived from the National Institute on Alcohol Abuse and Alcoholism (NIAAA) and the Centers for Disease Control and Prevention [41,42,43] in a format similar to how participants receive PNF intervention content (i.e., on a lab computer, with audio/visual synced components, and with think-aloud segments). 

### 1.7. Specific Aims and Primary Hypotheses 

AIM I: Examine main effects of the PNF intervention in reducing perceived alcohol use norms of other same-sex University of Southern California (USC) college students, individuals’ own self-reported drinking behavior, and individuals’ alcohol-related consequences. 

We hypothesized main effects of the PNF-Only and PNF-ATSS conditions on intervention efficacy such that participants in both intervention conditions would demonstrate greater reductions one-month post-intervention in perceived typical same-sex student norms (Hypothesis 1), alcohol use (Hypothesis 2), and alcohol-related consequences (Hypothesis 3), relative to control participants. Furthermore, given the high level of experimenter control in the current study and low probability of distractions, we anticipated no differences between the PNF-Only condition and the PNF-ATSS condition on intervention outcomes.

AIM II: Investigate mechanisms of change within the PNF intervention. 

We hypothesized that there would be indirect effects of ATSS codes on changes in perceived norms and alcohol use one-month post-intervention (Hypothesis 4). We further anticipated an indirect effect of reductions in perceived norms from baseline to immediately post-intervention (i.e., reductions in normative misperceptions) on changes in alcohol use one-month post-intervention (Hypothesis 5).

## 2. Materials and Methods

### 2.1. Participants and Recruitment

Male and female undergraduate students (*N* = 70, 51% female) ranging in age from 18 to 21 (*M* = 19.01 years), who had been issued a first offense citation for violating the campus alcohol policy, completed all aspects of this study. Their class standings were 50.0% Freshmen, 28.6% Sophomore, 18.6% Junior, 2.9% Senior. Students self-identified as White (64.3%), Asian (27.1%), and African American (8.6%). A third of the sample were members of a fraternity or sorority (32.9%), indicating a slight overrepresentation compared to the overall student body involved in fraternities/sororities (approx. 25%). Table 1 provides descriptive information for demographic variables as well as for baseline and follow-up measures of all outcome variables in the overall sample and by study condition.

This study was approved by the university Institutional Review Board (IRB) (Approval #UP-16-00499) and a certificate of confidentiality was issued by the National Institutes of Health. In coordination with USC Student Judicial Affairs, students were offered the choice to either participate in our research study or complete a separate alcohol awareness program through Student Affairs. The participation rate into our study comprised a relatively small portion of the total adjudicated student population for the academic year and thus, recruitment into our study was incidental. Participating in our study served to fulfill the students’ sanctions. Upon receiving confirmation of expressed interest in participation, that is, after students enrolled in our research study as an alternative sanction, participants were randomized to one of the three conditions using a 2:1:1 allocation ratio: PNF-ATSS, PNF-Only, or Control+ATSS. Students were given information regarding informed consent, the research protocol, the risks and benefits of participation, the voluntary nature of participation, measures to ensure confidentiality, how to complete the pre-intervention survey, and how to sign up for the lab-based portion of the study. Surveys were sent out and administered using Qualtrics online data collection software. Recruitment and retention were further bolstered by the study’s incentive structure ($40 for completing the one-month follow-up survey).

### 2.2. Procedures 

Recruitment, baseline (pre-intervention) assessment, the intervention, and one-month follow-up assessment took place across two semesters in the 2016–2017 academic year. All data were collected via self-report directly from participants. All interventions occurred within two to three weeks following completion of a baseline assessment. At the end of the intervention and while still in the laboratory, the participants completed a brief online post-intervention assessment of their normative perceptions. This was followed by instructions for how to complete the follow up assessment and receive the monetary incentive. 

#### 2.2.1. Baseline Assessment

After agreeing to participate in the study, each student received an email containing an electronic link to the online survey that served as the baseline assessment. Upon clicking on the link, the participant viewed the informed consent form and provided electronic consent before being directed to the survey itself. At the end of the survey, the participant was given instructions for how to sign up for the lab-based intervention and assessment. 

#### 2.2.2. PNF Intervention Protocol

Following completion of the baseline survey, participants arrived during their scheduled appointment for the lab portion of the study, were reconsented, and were given a brief introduction to the agenda. Next, participants were seated at a computer and presented with an example feedback slide to introduce the types of information that would subsequently be shown. The slide was built for the participant, one piece at a time, and pre-recorded audio guided the participant with an explanation about what each component of the slide represents. The audio was synced with visual objects and arrows pointing at each part of the feedback graph, as it was introduced, until it was a complete feedback slide. 

Participants then progressed through the normative feedback designated by their assigned condition. Both PNF conditions contained four individually tailored feedback slides (each slide referring to a different drinking behavior). Text and separate graphs, each including three bars, were used to present information regarding the average number of drinks per drinking occasion, the total number of drinks consumed per week, the maximum number of drinks consumed on one occasion in the past month, and binge drinking frequency for (a) one’s own drinking behavior, (b) their reported perceptions of a typical same-sex USC student, and (c) the self-reported same-sex USC college student drinking norms. The bottom of each respective slide contained source information for the data, noting that the information came from a large, representative sample of USC students surveyed in 2016 (also including the sample size for the survey).

The data used in constructing the electronic normative feedback graphs were derived from two sources. An individual’s perceived group norms and individual alcohol use were collected from responses on the baseline survey. The self-reported student drinking norms were obtained from an IRB-approved 2016 representative campus wide survey of over 800 students that the lead author conducted in coordination with the Wellness and Health Promotion Office at USC. After viewing the example feedback slide, participants were given an opportunity to ask any questions about the graphical feedback display. 

#### 2.2.3. PNF-ATSS Intervention Protocol

The ATSS is a flexible paradigm that allows for an in vivo, “online” assessment of the content of cognitions and affective dynamics in a semi-structured manner. In a review of numerous studies that utilized the ATSS paradigm, Davison et al. [36] provided support for the predictive, face, concurrent, and construct validity of the paradigm. They also note that the reliability of coding (Pearson’s product moment coefficient) can range between 0.75 and 0.86. This is an impressively high correlation coefficient that arguably depends not only on the quality of the codes used, but on the extent of the coders’ training. With an assessment tool like ATSS, psychometric issues are often brought up in response to the coding of data rather than the data themselves [44]. In the case of ATSS, validity and reliability, especially inter-rater reliability, are contingent on the coding scheme selected and the training of coders. To the degree that the coding scheme has relevance to the topic being discussed and inter-rater reliability is acceptable, ATSS yields valid and reliable data. We invested considerable efforts in these undertakings. In addition to the creator of the ATSS paradigm, high-achieving undergraduate research assistants were recruited to aid in the development and piloting of intervention materials during the summer of 2016 and continued to aid in all aspects of the study’s implementation, including coding of ATSS-generated qualitative information. 

As in the PNF-Only condition, participants arrived during their scheduled appointment for the lab portion of the study and were given a brief introduction to the agenda. This time, in addition to being presented with an example feedback slide to introduce the types of information that would subsequently be shown, participants also received instructions for the think-aloud portion of the intervention. We modeled the PNF on how complex scenarios are traditionally divided into short segments in the ATSS so that think-aloud data could be obtained as the PNF presentation unfolds. The experimenter informed the participants that as they listen and follow along to the slide presentation, they would periodically hear a bell tone, which would be followed by a 60-s pause in the presentation. During this pause, the participants were instructed to speak into a small microphone their immediate thoughts and feelings at that particular point of time in reaction to the graphical feedback unfolding in the presentation. Once it had been ensured that the participant understood the task at hand in the course of the practice trial, the experimenter then began to record the audio in the microphone, cued the main PNF presentation, and exited the room. The ATSS assessment was conducted twice during each of four PNF slides that were shown, for a total of eight think-aloud segments. When finished, the experimenter presented further instructions for the immediate post-intervention survey. 

#### 2.2.4. Control Condition

Both intervention conditions were compared to an active control condition, in which participants received alcohol-related psychoeducation based on information published by the NIAAA and the Centers for Disease Control and Prevention [41,42,43]. This presentation included information to educate participants about the risks associated with college drinking, the effects of alcohol intoxication, and how to recognize signs of alcohol poisoning. The presentation also “debunked” common myths about alcohol such as, “I can drive safely after a couple drinks” by presenting participants with contradictory facts, such as “Approximately half of all fatal car crashes in young adults between the ages of 18 and 24 involve alcohol”. To control for setting and interface, this information was presented in the same format as the two active conditions, a PowerPoint presentation with audio narration. To control for potential effects conferred by the ATSS think-aloud portion of the PNF intervention, participants in the control condition also participated in the think-aloud assessment during their presentation. An equal number of think-aloud opportunities were presented in both conditions. 

#### 2.2.5. Post-Intervention Assessment

Immediately following completion of the presentation within each respective condition, participants were asked to complete a brief online survey. To determine the ability of the PNF intervention to immediately correct normative perceptions, participants responded to the identical measure as assessed at baseline (Drinking Norms Rating Form, see below). 

### 2.3. Measures

Measures were carefully chosen to assess behavioral patterns and main outcome targets and were reported over the past week or month to reduce problems with retrospective recall of behaviors. To reduce the likelihood that reporting on normative estimates biased one’s own self-reported drinking, these measures were spaced far apart in the survey. 

#### 2.3.1. Demographic Information

Age, sex, ethnicity, class year, and Greek-membership affiliation were assessed.

#### 2.3.2. Alcohol Consumption

Participants completed the Daily Drinking Questionnaire (DDQ) [45], which is designed to assess typical daily alcohol consumption over a specific time period (past 30 days in the present study). Participants are asked to estimate the typical number of drinks consumed (based on a definition of a standard drink provided to participants) on each day of the week, which, when summed together, provide a composite variable of the total number of drinks consumed in a typical week. The DDQ has been commonly used in alcohol intervention studies and has been shown to be a reliable and valid measure of college student drinking [46,47]. 

#### 2.3.3. Alcohol-Related Consequences

Alcohol problems were assessed using the Rutgers Alcohol Problem Index (RAPI) [48]. The RAPI assesses the occurrence of 24 negative consequences resulting from one’s drinking over the past month (e.g., “Not able to do your homework or study for a test” and “Had withdrawal symptoms, that is, felt sick because you stopped or cut down on drinking”). Each item is rated on a scale from 1 to 4, with 1 indicating “never” and 4 indicating “more than 10 times”. A summed composite was formed for use in the study. 

#### 2.3.4. Perceived Drinking Norms

The format of the Drinking Norms Rating Form (DNRF) [49] mirrors that of the DDQ, except participants provide estimates of alcohol use for a particular reference group, in this case, a typical same-sex USC student. The DNRF perceived total weekly drinking was used as a primary outcome measure. The DNRF and modifications thereof have been used in numerous studies related to social norms and college student drinking. It has consistently demonstrated good prospective and concurrent validity and has good test-retest reliability [33].

### 2.4. ATSS Coding Strategy

PNF-ATSS think-aloud data and the control condition think-aloud data were recorded and analyzed iteratively using an a priori coding scheme. This coding scheme was created through several months of review of participant audiotapes and transcripts by the authors’ Lab for Cognitive Studies in Clinical Psychology, which included several graduate students and several undergraduate student research assistants. Multiple iterations of coding categories and code definitions were generated, and seven coding categories were ultimately selected based on observed themes that functioned as a continuum of factors hypothesized to relate to behavior change or the absence thereof: Sustain talk, skepticism, follow/neutral, believability, reflective analysis, positive surprise, negative surprise. Thirty to forty example statements were then extracted from transcripts as representative statements of specific scores within each coding category. The resulting coding manual explained in depth the seven coding categories by defining their characteristics (see below), describing how each code may appear within transcripts, and providing 10–20 scoring examples for each code.

1. Sustain talk, resistance, defensiveness, “digging in one’s heels”

This coding category captures statements referring to the person’s own arguments for not changing, for sustaining the status quo. They may minimize their own drinking behaviors/levels or give reasons why they do not need to change either their perceptions or their own behavior.

2. Discounting/skepticism/rejection of data

This coding category can be viewed as the opposite of the believability category. It captures statements of the various ways in which participants discount the data being presented. Examples include skepticism regarding the source or accuracy of the data, justification for their original beliefs, or anecdotal reflections of personal experience that lead them to reject the information they are viewing.

3. Follow/neutral

Statements within this category include simply restating or summarizing what participants are viewing. Tone and content reflect a degree of indifference to or disengagement from information being presented. Scores reflect the extent to which participants are simply following along with the presentation.

4. Believability of data

This coding category captures statements referring to the degree to which a participant believes the data that are presented. Statements may refer to the source of the data, the individual’s reports of own drinking or estimates of others’ drinking, or the self-reported group norm. This code is not to be scored unless there is explicit reference to the data in some fashion.

5. Reflective analysis & belief modification

This coding category refers to the extent to which participants acknowledge that a shift in perspective might be needed or attempt to actually modify existing beliefs in light of the information being presented. Statements include a thoughtful analysis of the data in which previously held beliefs/opinions/perspectives are reconsidered. Explanations may involve recalling anecdotal examples that justify and are consistent with what the participant is viewing or identifying specific reasons why original beliefs may be incorrect. Scored statements reflect a depth of processing that goes beyond simply summarizing what they are viewing (see, follow/neutral) or reflect a degree of self-exploration in which participants view their own behavior/perceptions or others’ behavior in a new light.

6. Surprise
  - The surprise (6a) coding category captures primarily positively valanced emotional expressions of surprise, shock, amazement, or astonishment in reaction to the presented data. It may involve an emotional/affective reaction to a difference between one’s perceived norm and the self-reported group norm.
  - The surprise (6b) coding category captures primarily negatively valanced emotional expressions of surprise, shock, amazement, or astonishment in reaction to the presented data. Coded (negative) reactions of surprise may often accompany skepticism of the data (e.g., “Wow, I definitely I do not believe those numbers.”). In this case, dual scores for both surprise (6b) and skepticism are warranted.

Each 60-s ATSS think-aloud segment of participants’ verbalizations was individually analyzed for the presence and intensity of the coding categories using a four-point Likert scale [0—not at all (complete absence of code); 1—slightly/somewhat (low presence of code); 2—moderately (moderate presence of code); 3—very (high presence of code; unequivocal endorsement)]. These ratings were recorded for use in the reliability analyses (see below for description of interrater reliability). The two coders were highly trained to ensure adequate reliability and weekly coding meetings were held to protect against coder drift. 

To resolve minor discrepancies between raters and arrive at a final score, a third-rater adjudication procedure was used. Several transcripts were rated each week by the two independent coders. Then during weekly coding meetings, each transcript was reviewed and the rationale behind code assignment was discussed as a group by the two coders and the lead author. The ratings for all codes within all segments made by the two coders were reviewed with the lead author until consensus was reached between all three coders based on the definitions outlined in the coding manual. These final scores were then used to generate a single summary score for each code, for each participant, by summing the scores for a particular code across all segments. The same coding procedure was applied to the control condition think-aloud segments. 

### 2.5. Interrater Reliability for Codes

To evaluate the interrater reliability for the seven ATSS codes (sustain talk, skepticism, follow/neutral, believability, reflective analysis, positive surprise, negative surprise), intraclass correlation coefficients (ICCs) were calculated based on the coding scores obtained from two independent RA coders after being summed across all segments. ICCs are typically used to assess the consistency of continuous measurements and/or ratings made by two or more observers reporting on the same quantity [50]. Values reported in the tables are based on the two-way mixed model (average measures). This model was chosen due to the fact that the two ratings came from the same two coders for each participant [51]. Table 2 reports values using the absolute agreement criterion, while Table 3 uses the consistency criterion.

Cicchetti [52] offers the following guidelines for interpretation of ICC inter-rater agreement measures: Less than 0.40—poor; Between 0.40 and 0.59—fair; Between 0.60 and 0.74—good; between 0.75 and 1.00—excellent. The results of the reliability analysis indicate that all seven of the coding categories within the PNF-ATSS condition fell in the ‘excellent’ reliability range, while reliabilities in the control condition ranged from the ‘good’ to ‘excellent’ range. The lack of noticeable differences between the ICC values based on absolute agreement and those based on consistency indicates that the coding disagreements were unsystematic (i.e., there was no main effect of coder). 

## 3. Results

### 3.1. Missing Data, Outliers, and Randomization Check 

In the overall sample, 94.8% of the participants assessed at baseline (*N* = 77) completed the follow-up survey one month later (*N* = 73). The four non-completers were evenly split between the PNF-Only and the control conditions. No differences were observed on primary study variables between the students who dropped out and those who remained in the study through follow-up (all *p*s > 0.05). 

Cook’s distance was calculated for the main outcomes to determine the presence of outliers prior to data analysis. Three influential data points were observed (Cook’s d > 1.3). Upon closer examination, the three participants responded incorrectly to two attention-control questions embedded in the survey and exhibited a pattern of random responding. Thus, they were excluded from all analyses. The final overall sample used in analyses was *N* = 70. For analyses, we did not impute missing values but rather used all available data for each specific analysis. Thus, minor discrepancies in degrees of freedom reflect missing data. 

Kruskal-Wallis tests for independent samples were conducted to evaluate baseline differences among the three conditions (PNF-ATSS, PNF-Only, control). Results revealed no significant differences among the three conditions on weekly drinking, χ^2^(2) = 1.82, *p* = 0.40, alcohol-related consequences χ^2^(2) = 0.09, *p* = 0.96, or perceived weekly drinking norms χ^2^(2) = 0.29, *p* = 0.86, whereby supporting the equivalency of intervention and control groups at baseline. 

### 3.2. Preliminary Analyses

Means and standard deviations for all of the primary outcomes are displayed in Table 1. Bivariate correlations were conducted between the primary variables of interest (Total Drinks Per Week, Perceived Weekly Peer Drinking Norms, and Alcohol-Related Consequences), pre- and post-intervention. One-sample *t*-tests determined whether a significant difference was present between students’ perceptions concerning other same-sex USC students’ alcohol use behaviors and the self-reported drinking behaviors of students of their same sex. 

#### 3.2.1. Correlations

Bivariate correlations were computed separately for males and females on the main variables (see Table 4). Of particular interest, among males, Time 1 weekly drinking was positively correlated with Time 1 perceived drinking norms of other male USC students and Time 2 weekly drinking. Among females, Time 1 weekly drinking positively correlated with Time 1 alcohol-consequences and Time 2 weekly drinking, but not with perceived drinking norms of other female USC students at Time 1.

#### 3.2.2. Misperceptions: Perceived Peer Drinking vs. Self-Reported Student Drinking

Male participants’ perceived norms were approximately double that of the self-reported male student drinking norms at USC, across all four drinking variables (see Table 5): Average number of drinks per occasion, total number of drinks per week, maximum number of drinks consumed at any one time in the past month, and binge drinking frequency in the prior two weeks. Similarly, female participants’ perceived norms were more than double that of the self-reported female student drinking norms at USC, across the same four drinking variables (see Table 5).

### 3.3. AIM I: Analytic Plan 

One-way analyses of covariance (ANCOVA) were used to examine overall main effects of the interventions with: (a) post-intervention outcome as the dependent variable (Total Drinks Per Week, Perceived Weekly Peer Drinking Norms, and Alcohol-Related Consequences); (b) the control and two intervention groups as levels of the independent variable; and (c) the baseline outcome variable as the covariate. Follow-up tests were conducted to evaluate pairwise differences among the estimated marginal means for intervention conditions. Please refer to Supplementary Material Document S1: Assumptions of the General Linear Model, for a description of the normalization procedure used on the main outcomes and coding category scores.

### 3.4. AIM I: Intervention Main Effects

#### 3.4.1. Weekly Drinking

The ANCOVA examining post-intervention weekly drinking revealed a marginally significant main effect of condition after controlling for baseline weekly drinking, *F*(2, 69) = 2.98, *p* = 0.05, η_p_^2^ = 0.08. The results showed that participants in the PNF-ATSS condition (*M* = −0.138) drank significantly fewer drinks per week post-intervention, controlling for baseline drinking, than participants in the PNF-Only condition (*M* = 0.303), but not less than participants in the control condition (*M* = −0.009). 

#### 3.4.2. Alcohol-Related Consequences 

The ANCOVA examining post-intervention alcohol-related consequences revealed a significant main effect of condition after controlling for baseline consequences, *F*(2, 68) = 3.23, *p* = 0.04, η_p_^2^ = 0.09. The results showed that participants in the PNF-ATSS condition (*M* = −0.067) and those in the PNF-Only condition (*M* = −0.123) reported fewer alcohol consequences post-intervention, controlling for baseline levels, than participants in the control condition (*M* = 0.409). 

#### 3.4.3. Perceived Weekly Drinking Norms

The ANCOVA examining post-intervention perceived peer drinking norms revealed a significant main effect of condition after controlling for baseline perceived norms, *F*(2, 64) = 3.70, *p* = 0.03, η_p_^2^ = 0.11. The results showed that participants in the PNF-ATSS condition (*M *= −0.061; marginal effect *p* = 0.05) and those in the PNF-Only condition (*M* = −0.395) held lower perceived weekly drinking norms post-intervention, controlling for baseline perceived norms, than participants in the control condition (*M* = 0.471). 

### 3.5. AIM II: Analytic Plan

Prior to conducting tests of indirect effects, correlations examined bivariate relationships between the seven coding categories and changes from baseline to one-month follow-up on drinking and perceived norms. Change scores for perceived norms and drinking were calculated by subtracting baseline scores from follow-up scores on the two variables, respectively. Positive change scores indicated increases in perceived norms and/or drinking, whereas negative change scores indicated decreases in these constructs. Next, a series of independent-samples t-tests were conducted to compare mean scores on the various ATSS coding categories between the PNF-ATSS and control conditions. 

An SPSS macro developed by Andrew Hayes [53,54] was used to test for possible indirect effects. The predictor variable in all the models was dummy-coded intervention condition, (0 = Control, 1 = PNF-ATSS). The criterion variable was total drinks per week or perceived norms at follow-up. The mediator was the respective designated ATSS code. Steps for examining indirect effects were based on Kenny, Kashy, and Bolger [55]. Path *c* coefficients are reported in Table 8 note, but the significance of path *c* was not required for establishing mediation [56]. Path *c*’ coefficients are presented in Table 8 in the presence of a significant indirect effect. Indirect effects were tested using a percentile bootstrap estimation approach with 5000 samples [57,58] and confidence intervals reported are “percentile” intervals, which do not involve a bias correction [59,60]. 

### 3.6. AIM II: Correlations and Tests of Mean Differences

First, correlations were computed separately for PNF-ATSS and control conditions on pre/post change scores for perceived norms and drinking along with scores on all seven coding categories (see Table 6). Of particular interest, among control participants, changes in weekly drinking were positively correlated with changes in perceived norms, such that as norms decreased, drinking decreased. Lower scores on negative surprise were correlated with decreases in drinking. Also, among control participants, increases in perceived norms were correlated with higher scores on the follow/neutral code, whereas more believability was associated with decreases in drinking. 

Among participants in the PNF-ATSS condition, changes in weekly drinking were positively correlated with changes in perceived norms, such that as norms decreased, drinking decreased (and vice versa). Higher scores on follow/neutral were associated with increases in drinking, whereas higher scores on skepticism were found to be related to decrease in drinking. 

Also, among PNF-ATSS participants, change scores in perceived norms were negatively correlated with skepticism and with negative surprise, such that more skepticism and greater negative surprise were associated with decreases in perceived norms, an unexpected finding. Conversely, higher scores on follow/neutral were associated with increases in perceived norms.

Next, a series of independent-samples t-tests were conducted to compare mean scores on the various ATSS coding categories between PNF-ATSS and control conditions (See Table 7). Differences between the conditions on four of the seven coding categories emerged as significant. Participants in the PNF-ATSS condition were found to articulate more skepticism and negative surprise, but less follow/neutral and believability, than those in the control condition.

### 3.7. Aim II: Evaluating Indirect Effects of ATSS Codes on Drinking

As shown in Table 8, a significant indirect effect revealed that being in the PNF-ATSS condition was associated with lower levels of neutrality regarding the intervention content, and lower levels of neutrality, in turn led to lower drinking at follow-up. None of the other models supported the indirect effect hypothesis.

### 3.8. Evaluating Explanatory Mechanism of ATSS Codes on Perceived Norms

As shown in Table 8, significant indirect effects were observed for follow/neutral and believability. Receiving the PNF-ATSS intervention was associated with lower levels of neutrality regarding the intervention content, and lower levels of neutrality, in turn led to lower perceived norms at follow-up. Receiving the PNF-ATSS intervention was also associated with lower levels of believability, yet lower levels of believability led to higher perceived norms at follow-up. 

### 3.9. Evaluating Indirect Effect of Changes in Perceived Norms on Drinking

Regression analysis was also used to investigate the hypothesis of an indirect effect from changes in perceived norms immediately post-intervention to one-month post-intervention drinking. The predictor variable in all the models was dummy-coded intervention condition, (0 = Control, 1 = PNF-ATSS). The criterion variable was total drinks per week at follow up. The mediator variable was changes in perceived norms. We created change scores for perceived norms by subtracting baseline perceived norms from perceived norms collected immediately post-intervention and while still in the lab. Positive change scores indicated increases in perceived norms, whereas negative change scores indicated decreases in perceived norms. 

Results indicated that while controlling for baseline drinking, intervention condition was not a significant predictor of drinking at 1-month follow-up, B = −0.13, SE = 0.18, *p* = 0.46, but was found to predict changes in norms immediately post-intervention, B = −4.52, SE = 1.64, *p* = 0.008. With baseline drinking and intervention condition included in the model, however, changes in norms were not found to be a significant predictor of post-intervention drinking, B = 0.01, SE = 0.02, *p* = 0.45. These results do not support the indirect effect hypothesis. 

## 4. Discussion

The present study aimed to broaden our understanding of alcohol interventions targeting groups at risk for heavy and problematic alcohol use by applying a novel adaptation of a cognitive assessment strategy (ATSS) to identify potential mechanisms of change. Utilizing prior research to inform intervention content, this study included three intervention conditions: one condition involved viewing standard PNF without ATSS and the second condition included PNF-ATSS. Both intervention conditions were compared to each other and to an active control condition which received alcohol-related psychoeducation derived from the NIAAA and the Centers for Disease Control and Prevention and that also included the ATSS think-aloud component. 

Previous studies on the efficacy of PNF among adjudicated students were performed in the context of web-based multicomponent interventions that included PNF among other intervention content, rather than stand-alone PNF, making it difficult to ascertain which components were most responsible for reductions in drinking. Findings from a recent meta-analysis [28] suggest that computer-delivered stand-alone PNF is a promising prevention approach for college student drinking, but has minimal impact on alcohol-related consequences. Overall, effect sizes observed in the meta-analysis were small but significant for alcohol use and less than small for alcohol-related consequences. Importantly, the eight studies reviewed in the meta-analysis did not target adjudicated student samples. The present study evaluated the efficacy of stand-alone PNF provided to adjudicated students in a lab setting compared to an active control condition.

As anticipated, and consistent with prior studies involving non-adjudicated [61] and adjudicated student samples [7], the overall adjudicated student sample held large misperceptions of drinking behavior self-reported by fellow same-sex USC students, overestimating by at least double across four different drinking behaviors. 

AIM I of the current study examined the main effects of the PNF intervention in reducing perceived alcohol use of other same-sex USC college students, individuals’ own drinking behavior, and individuals’ alcohol-related consequences. The findings completely supported Hypothesis 1. Students in both PNF conditions reported a significant reduction in their misperceptions at the 30-day follow-up, relative to students in the control condition, who were not found to have significantly reduced their misperceptions. Moreover, there were no observed differences between the two PNF conditions. 

With respect to self-reported drinking, the findings partially supported Hypothesis 2. The results showed that participants in the PNF-ATSS condition drank significantly fewer drinks per week post-intervention, controlling for baseline drinking, than participants in the PNF-Only condition, but not less than participants in the control condition. No differences were found between the control and PNF-Only conditions. 

Although reducing misperceived drinking norms is generally considered a pre-requisite to accompanying changes in drinking, this did not occur with the PNF-Only condition. If PNF alone was sufficient to produce behavior change, then alcohol use should have been reduced in this group as well, since there was an intervention effect on reducing overestimated normative perceptions. Yet the intervention did not reduce self-reported alcohol use. In fact, those students who received PNF without ATSS slightly increased drinking (albeit non-significantly), while control participants slightly decreased drinking, though also non-significantly. 

This pattern of findings, while at first somewhat counterintuitive, may relate to the shared “think-aloud” methodology inherent in the PNF-ATSS and control participants’ interventions. It is possible that stopping periodically to reflect aloud occasioned greater attention to intervention material, thereby facilitating greater internalization of the material presented relative to those who were only presented PNF without such additional opportunities/prompts. According to social norms theory, normative influence will lead to behavior change only when “highlighted prominently in consciousness” [62] (p. 597). That ATSS may encourage better memory of source material and thereby contribute to an intervention effect is an interesting area for future research. Hitherto, ATSS has been considered as strictly an assessment paradigm; its possible “Heisenberg effect” properties have not been a focus of research.

Finally, and in concordance with the findings on perceived norms, Hypothesis 3 was completely supported. Students in both PNF conditions reported a significant reduction in their alcohol-related consequences at the one-month follow-up relative to students in the control condition, who were not found to have a significant decrease in alcohol problems. No differences were observed between the two PNF conditions, despite the fact that only participants from the PNF-ATSS condition were found to reduce drinking. 

Focusing on the shared nature of their intervention content, receiving normative feedback appears to be uniquely associated with drinking in a less risky fashion. Perceived norms have been shown to predict a composite of alcohol-related consequences even after controlling for level of consumption [63]. This is an important finding given the now widely adopted focus on harm-reduction as a more realistic goal than abstinence for problematic drinking behaviors among young adults [64,65]. A primary reason for devoting resources aimed at understanding and intervening in student drinking is the alcohol-related problems that are experienced by drinkers and, indirectly, their non-drinking peers and communities. Most current alcohol treatment programs for college students include moderate drinking and harm reduction as their primary goals. The sought-after reduction in harm, however, is nearly always viewed as a function of reductions in alcohol use as the primary outcome variable. It is, therefore, clinically significant that the results of this study support PNF as a means of reducing harm in the absence of reductions in drinking (as is the case with the PNF-Only participants). 

### 4.1. ATSS and Mechanisms of Change 

AIM II of the study entailed a wider investigation of potential change mechanisms involved in intervention efficacy. One of this study’s authors along with other writers argued many years ago that research concerning itself solely with the comparison of treatments to one another takes clinical psychological scientists away from what could and perhaps should be our primary mission, namely, to explain *why* a given intervention effects beneficial change [66,67,68,69,70,71,72,73]. Understanding change mechanisms can enhance attainment of a desirable treatment goal at the individual or population level. The ATSS procedure used in this study provided unique insight into the phenomenology of college students being exposed to intervention content by capturing moment-to-moment thoughts and feelings throughout the process. 

Themes derived from preliminary analysis of audio recordings collected during think-aloud segments were translated into seven coding categories, each of which was rated as being present to a certain degree (as described below) following formal coding procedures. The “sustain talk” and “skepticism” codes were anticipated to act as barriers to changes in beliefs and drinking behavior. When comparing the PNF-ATSS to the Control condition, no significant difference was found between the two conditions on the degree of sustain talk evidenced across the intervention. However, participants in the PNF-ATSS condition (*M* = 3.16) were found to articulate greater skepticism of the content than those in the Control condition (*M* = 1.29). 

The follow/neutral code was expected to reveal little in the way of understanding a participant’s cognitive or emotional reactions to the intervention information being presented. The coding manual suggested that the ambiguity of the articulated statements that fell into this category did not allow for inferences regarding the extent to which a person believed the data or what change, if any, the information would bring about in the individual. Participants in the Control condition (*M* = 5.86) exhibited higher scores on follow/neutral than those in the PNF-ATSS condition (*M* = 3.06), reflecting, perhaps, the novelty of more analytic information contained in PNF. Interestingly, among both Control and PNF-ATSS participants, higher scores on the follow/neutral code were predictive of less movement toward reductions in perceived norms post-intervention, and the same was true for PNF-ATSS participants with respect to drinking. 

There were also significant indirect effects from the intervention to drinking and perceived norms outcomes via follow/neutral, in support of Hypothesis 4: being in the PNF-ATSS condition was associated with lower levels of neutrality regarding the intervention content, and lower levels of neutrality, in turn led to lower drinking and lower perceived norms at follow-up. These findings corroborate a recent study in which, during a brief MI-delivered motivational intervention for alcohol use targeting adjudicated students, follow/neutral utterances (as coded by the Motivational Interviewing Skill Code 2.0; [74]) were associated with increases in drinking at follow-up [75]. It is likely that, although less subtle than sustain talk or skepticism, following along (“uh huh”) to intervention content may represent resistance rather than engagement. Indeed, tone and content of the articulations within the current sample reflected a degree of indifference to or disengagement from the intervention content.

Progressing forward on the continuum of factors hypothesized to relate to behavior change are believability and reflective analysis. Participants in the PNF-ATSS condition articulated weaker explicit statements of believability (*M* = 3.10) compared to those in the Control condition (*M* = 6.64). In addition, among control participants, more believability was bivariately associated with change scores representing decreases in drinking. The more they believed the psychoeducational materials, the greater the change. A significant indirect effect emerged from the intervention on perceived norms via believability, also in support of Hypothesis 4: being in the PNF-ATSS condition was associated with lower levels of believability regarding the intervention content. The lower the level of believability regarding the content, the higher perceived norms were at follow-up. This finding highlights the importance of making PNF content (as well as other psychoeducational content) as believable as possible in order to maximize intervention efficacy. 

Reflective analysis was the final ‘cognitive’ code on the continuum that we anticipated to occasion a greater shift in post-intervention outcomes. Reflective analysis referred to the extent to which participants shifted their beliefs or perspectives in light of the information presented. Contrary to expectations, no differences were found between the two conditions on this construct. Participants in both conditions articulated relatively high degrees of self-reflection throughout the presentations. Although reflective analysis was found to negatively predict post-intervention norms (though not drinking), the lack of an intervention effect on this construct negated a test of indirect effects. 

The final code applied to the ATSS data was surprise. A code for “positive surprise” attempted to capture more positively valanced expressions of surprise, shock, or amazement, in reaction to the presented data, while “negative surprise” captured primarily negatively valanced emotional expressions of surprise or shock. No significant findings emerged with respect to positive surprise. Although negative surprise was present only to a relatively small degree for both groups, participants in the PNF-ATSS condition (*M* = 1.13) were significantly higher in negative surprise than those in the Control condition (*M* = 0.14). For control participants, less negative surprise was significantly associated with change scores representing decreases in drinking. Among PNF-ATSS participants; however, more negative surprise was associated with decreases in perceived norms. 

With respect to Hypothesis 5, results did not support an expected indirect effect of reductions in perceived norms from baseline to immediately post-intervention on changes in alcohol use one-month post-intervention. Despite the presence of large normative misperceptions of peers’ drinking, among males, pre-intervention weekly drinking was positively correlated with perceived drinking norms of other male USC students, but among females, this finding did not emerge. Such a phenomenon may partially account for the smaller main effects between study conditions as well as the null finding for an indirect effect that has been demonstrated in other studies [31,34,76]. 

### 4.2. Limitations

Careful consideration was given to the ways in which the research design strengthens generalizability and reliability of conclusions to be drawn from the findings. Potential drawbacks nonetheless remain. The nature of sensitive information such as alcohol use often comes with attendant concern about the validity of self-report in this population. There is, however, a substantial body of research supporting the validity of self-report measures of alcohol consumption [77,78,79]. Other external sources of information, such as collateral information, are not readily available or practical for assessing college drinking. Because confidentiality enhances the reliability and validity of self-report data [80], participants were continuously reminded that all data were kept in strict confidence. Despite attempts to assure the students that their responses to the questionnaires were completely confidential, there is always the possibility that perceived coercion to complete a mandated sanction resulted in underreporting on drinking indicators, though prior research has found little evidence of intentional bias for alcohol use reporting among adjudicated students [81]. Relatedly, social desirability bias was not measured in this study and that presents another potential limitation, as it has been found to be a significant predictor of changes in drinking over time in some social norms intervention studies [82]. 

Secondly, students were given the option to participate in this study or receive the standard sanction, AlcoholEdu^TM^, and it is unknown how the selected sample may have differed in baseline characteristics from those who did not choose to participate. Comparatively small samples sizes within two of the study conditions may have interacted with selection effects to produce a pattern of findings that may differ if participation was mandated for all adjudicated students. 

Another potential confounding factor with respect to data validity is that adjudicated students may have already made initial reductions in drinking before volunteering for our research study due to having received a sanction [83]. This outcome would undoubtedly be favorable to student judicial affairs personnel but may have an unseen impact on some of the statistical relationships between constructs. Having said that, this study still produced positive change for many participants despite the potential effects of the sanctioning process, also surely of import to university personnel. 

The intervention’s effects were measured for a relatively brief follow-up period of 30 days, consistent with prior studies using PNF with adjudicated students [7,34]. The effects of web-based personalized feedback have been shown to last for up to 6 months in college students [84]. Future longitudinal research could examine the degree to which stand-alone PNF may attenuate drinking among adjudicated college students over longer follow-up periods of at least 6 months. Lastly, the pattern of findings observed in the current study may not translate as reliably in other non-U.S. countries. Local drinking cultures elsewhere may differ in the degree to which young people care about what others around them do, relative to their own behavioral norms (see social comparison theory, [85]). 

### 4.3. Strengths and Future Directions

Despite the potential drawbacks, the results of this study have important implications for brief intervention programs targeting adjudicated college students and for public health interventions more broadly. Adjudicated students remain a high-risk population for problematic drinking on college campuses. Studies targeting change mechanisms have been underemphasized in college-student drinking intervention literature: the functional importance of processes of change for many brief interventions has not been sufficiently well-established. More broadly, associations that certify evidence-based intervention methods, such as Division 12 (clinical psychology) of the American Psychological Association, have failed to require evidence of processes of change linked to the underlying theoretical model and procedures deployed [86]. 

The current study presents a proof of concept for an adapted ATSS think-aloud methodology as a clinical science intervention tool. The ability to capture in real-time cognitions and affect in response to intervention content provides a fertile opportunity to specify the processes of change linked to that intervention for particular problems, persons, and contexts. This methodology could also prove to be of utility when piloting larger and more complex public health interventions, by allowing researchers to better understand how participants cognitively navigate the intervention content and how such cognitions relate to outcomes. Among the ATSS’ advantages over other modes of assessment such as questionnaires and interviews are its situational specificity, investigator control, unconstrained response format, immediacy of assessment, unlimited choice of data coding approaches, empirical flexibility, detection of group-specific cognitive-affective differences, and the flexibility to study the cognitive aspects of a wide range of problems, including novel or sensitive domains of inquiry [35,37]. 

The present study also benefitted from a large sample size for the campus-wide survey documenting accurate drinking norms of the student body. Future research may wish to document norms among adjudicated students as well to explore whether including normative feedback pertaining to this subgroup may confer additive effects to more general student normative feedback as part of the sanctioning intervention. 

## 5. Conclusions

Though PNF may be limited in clinical significance as a stand-alone intervention, the observed effects on drinking are clinically relevant when PNF is examined from a public health perspective as an approach for intervening with problematic or higher-risk students, such as those who have been cited for violating their campus alcohol policy. These results add to the literature examining stand-alone PNF by describing the efficacy of the intervention to reduce alcohol-related outcomes among adjudicated college students and exploring explanatory mechanisms associated with efficacy using a novel cognitive assessment approach—the Articulated Thoughts in Simulated Situations think-aloud paradigm. Since cognitive processes are dynamic rather than static, the paradigm is an ideal assessment approach for detecting ongoing cognition under the conditions in this study and potentially in other public health intervention research. Such an application can assist interventionists in designing, piloting and choosing prevention strategies that will be maximally effective, while setting the stage for the adoption of think-aloud methodology as a clinical science tool for not only assessment, but potentially as a contributor to change itself.

## Figures and Tables

**Table 1:** Descriptive statistics for demographics characteristics and primary outcomes by study condition.

Variable	Overall (*N* = 70)		PNF-ATSS (*N* = 37)		PNF (*N* = 17)		Control (*N* = 16)	
	% (*N*)	M (SD)	% (*N*)	M (SD)	% (*N*)	M (SD)	% (*N*)	M (SD)
Age (Years)		19.01 (0.96)		19.08 (1.01)		18.82 (0.88)		19.06 (0.93)
Sex								
Male	51.4 (36)		54.1 (20)		41.2 (7)		43.8 (7)	
Female	48.6 (34)		45.9 (17)		58.8 (10)		56.3 (9)	
Ethnicity								
Hispanic	17.1 (12)		18.9 (7)		70.6 (12)		0 (0)	
Non-Hispanic	82.9 (58		81.1 (30)		29.4 (5)		100 (16)	
Race								
Asian	27.1 (19)		24.3 (9)		35.3 (6)		25.0 (4)	
Black or African American	8.6 (6)		8.1 (3)		5.9 (1)		12.5 (2)	
White or Caucasian	64.3 (45)		67.6 (25)		58.8 (10)		62.5 (10)	
Greek-Affiliation								
Greek Member	32.9 (23)		32.4 (12)		29.4 (5)		37.5 (6)	
Non-Greek Member	67.1 (47)		67.6 (25)		70.6 (12)		62.5 (10)	
Baseline Outcomes								
Weekly Drinks		8.32 (5.51)		9.14 (5.82)		6.74 (5.13)		8.13 (5.10)
Negative Consequences		30.13 (5.27)		30.11 (5.68)		29.29 (3.50)		31.06 (5.98)
Perceived Weekly Drinking		11.26 (5.62)		11.19 (5.52)		12.09 (7.16)		10.56 (4.00)
Follow-up Outcomes								
Weekly Drinks		6.91 (5.16)		6.78 (5.02)		7.24 (5.45)		6.88 (5.49)
Negative Consequences		28.80 (5.34)		28.00 (4.27)		27.63 (3.56)		31.81 (7.76)
Perceived Weekly Drinking		7.01 (4.06)		6.94 (4.35)		5.50 (3.17)		8.67 (3.75)

Note. There were no significant differences by intervention condition for demographics or outcome variables at baseline. PNF-ATSS: Personalized Normative Feedback-Articulated Thoughts in Simulated Situations.

**Table 2:** Interrater reliability using Intraclass Correlation Coefficients (ICCs) based on absolute agreement criterion.

Code	PNF-ATSS	Control
Sustain	0.99	0.64
Skepticism	0.98	0.97
Follow/Neutral	0.95	0.83
Believability	0.98	0.76
Reflective Analysis	0.99	0.79
Positive Surprise	0.97	0.66
Negative Surprise	0.96	0.90

**Table 3:** Interrater reliability (ICCs) based on consistency agreement criterion.

Code	PNF-ATSS	Control
Sustain	0.99	0.63
Skepticism	0.98	0.97
Follow/Neutral	0.95	0.82
Believability	0.98	0.82
Reflective Analysis	0.99	0.79
Positive Surprise	0.97	0.66
Negative Surprise	0.96	0.90

**Table 4:** Correlation matrix of variables for males (*n* = 34) and females (*n* = 36).

Measure Number	Measure	Mean (SD) Males	Mean (SD) Females	1	2	3	4	5	6
1	T1 Weekly Drinking	8.07 (6.16)	8.56 (4.90)	-	0.16	0.60 ***	0.75 ***	0.00	0.41 **
2	T1 Perceived Drinking Norm	11.72 (5.65)	10.83 (5.63)	0.32 *	-	0.22	0.25	0.19	0.04
3	T1 Alcohol Consequences	29.62 (5.27)	30.61 (5.30)	0.29	−0.01	-	0.43 **	0.04	0.70 ***
4	T2 Weekly Drinking	7.32 (5.88)	6.53 (4.43)	0.73 ***	0.08	0.13	-	0.01	0.36 *
5	T2 Perceived Drinking Norm	8.69 (4.34)	5.47 (3.13)	0.24	0.12	−0.29	0.45 **	-	0.35 *
6	T2 Alcohol Consequences	28.42 (5.56)	29.14 (5.18)	0.22	−0.17	0.70 ***	0.33 *	−0.16	-

Note. T1 refers to pre-intervention scores. T2 indicates post-intervention scores. Correlations for males are below diagonal, correlations for females are above diagonal. * *p* < 0.05; ** *p* < 0.01; *** *p* < 0.001.

**Table 5:** Students’ perceptions of typical same-sex USC student compared to students’ actual drinking.

Descriptive Norm	Perception of Typical Male Student (*n* = 38)	Self-Reported Drinking (*n* = 259)	*t* Value	Perception of Typical Female Student (*n* = 37)	Self-Reported Drinking (*n* = 379)	*t* Value
	M (SD)			M (SD)		
Average Number Drinks Per Occasion	4.54 (1.59)	2.76	6.67 ***	3.81 (1.37)	2.00	7.91 ***
Total Number Drinks Per Week	11.72 (5.65)	6.53	5.36 ***	10.83 (5.63)	4.33	6.93 ***
Maximum Number of Drinks	7.18 (2.70)	4.63	5.50 ***	5.58 (1.84)	2.95	8.58 ***
Binge Drinking Frequency Two Weeks	2.15 (1.60)	0.95	4.37 ***	2.06 (1.29)	0.77	6.00 ***

*** *p* < 0.001.

**Table 6:** Correlation matrix of outcome changes and coding categories for control (*n* = 14) and PNF-ATSS (*n* = 31).

Measure Number	Measure	1	2	3	4	5	6	7	8	9
1	Change in total drinks per week	-	0.38 *	0.08	−0.57 **	0.36 *	0.22	−0.12	0.20	−0.31
2	Change in perceived norms	0.56 *	-	0.19	−0.48 **	0.44 *	0.14	−0.28	−0.05	−0.39 *
3	Sustain talk	−0.41	−0.39	-	0.32	−0.29	−0.16	−0.45 *	−0.32	0.11
4	Skepticism	−0.52	−0.42	0.20	-	−0.29	−0.41 *	−0.29	−0.33	0.63 **
5	Follow/Neutral	0.31	0.66 *	−0.36	−0.46	-	−0.08	−0.36 *	0.03	−0.05
6	Believability	−0.31	−0.69 **	0.15	0.51	−0.61 *	-	0.27	0.21	−0.49 **
7	Reflective analysis	−0.01	0.22	−0.10	0.13	−0.53	−0.03	-	0.39 *	−0.25
8	Positive surprise	0.11	−0.12	−0.36	−0.38	0.06	0.25	−0.36	-	−0.05
9	Negative surprise	0.68 **	0.15	−0.14	−0.20	0.08	−0.20	−0.11	0.16	-

Note. Correlations for Control are below diagonal, correlations for PNF-ATSS are above diagonal. * Correlation is significant at the 0.05 level (2-tailed). ** Correlation is significant at the 0.01 level (2-tailed).

**Table 7:** Mean differences on coding categories as a function of intervention condition.

ATSS Coding Category	PNF-ATSS (*n* = 31)		Control (*n* = 14)		Independent *t*
	Mean	SD	Mean	SD	
Sustain Talk	3.26	5.08	1.29	2.67	−1.36
Skepticism	3.16	3.71	1.29	1.86	−2.26 *
Follow/Neutral	3.06	2.59	5.86	4.35	2.23 *
Believability	3.10	2.53	6.64	3.89	3.12 **
Reflective Analysis	6.42	4.37	4.64	4.24	−1.27
Positive Surprise	2.00	2.46	2.00	1.84	0.00
Negative Surprise	1.13	1.61	0.14	0.53	−3.06 **

Note. * *p* < 0.05. ** *p* < 0.01.

**Table 8:** Regression path coefficients and bootstrapped indirect effect of treatment condition on weekly drinking and perceived norms. through ATSS codes.

Mediators	*a*			*b*			*c*’			Indirect Effect		
Weekly Drinking (Outcome) ^1^	Beta	SE	*p*	Beta	SE	*p*	Beta	SE	*p*	Beta	SE	CI
Sustain Talk	0.38	0.23	0.10	−0.05	0.13	0.69						
Skepticism	0.49	0.26	0.06	−0.33	0.10	0.002						
Follow/Neutral	−0.72	0.27	0.01	0.23	0.10	0.03	0.10	0.19	0.59	−0.16	0.07	[−0.30, −0.03]
Believability	−0.92	0.26	0.001	−0.02	0.11	0.87						
Reflective Analysis	0.48	0.31	0.13	−0.08	0.09	0.42						
Positive Surprise	−0.07	0.28	0.82	0.17	0.10	0.09						
Negative Surprise	0.50	0.23	0.03	−0.09	0.13	0.49						
Perceived Norms (outcome) ^2^												
Sustain Talk	0.37	0.27	0.17	0.06	0.19	0.77						
Skepticism	0.45	0.25	0.08	−0.23	0.20	0.26						
Follow/Neutral	−0.62	0.28	0.04	0.48	0.16	0.005	−0.12	0.31	0.71	−0.30	0.18	[−0.71, −0.01]
Believability	−1.06	0.28	0.001	−0.44	0.17	0.01	−0.88	0.35	0.02	0.46	0.21	[0.10, 0.94]
Reflective Analysis	0.39	0.32	0.23	−0.35	0.15	0.02						
Positive Surprise	−0.02	0.31	0.96	−0.17	0.17	0.30						
Negative Surprise	0.51	0.21	0.02	0.06	0.24	0.82						

Note. Indirect effects were only evaluated in the presence of significant paths *a* and *b*. ^1^ Path *c* coefficients controlling for baseline drinking: B = −0.06, SE = 0.19, *p* = 0.74. ^2^ Path *c* coefficients controlling for baseline perceived norms: B = −0.41, SE = 0.32, *p* = 0.20.

## Supplementary Materials

The following are available online at https://www.mdpi.com/1660-4601/17/3/690/s1, Document S1: Assumptions of the General Linear Model.
