# Supplementary material for: Cognitive-Affective Change Mechanisms in Personalized Normative Feedback via the Articulated Thoughts in Simulated Situations Paradigm
Source: Int J Environ Res Public Health. 2020 Jan 21;17(3):690. doi: 10.3390/ijerph17030690 (PMC7038020; doi:10.3390/ijerph17030690)
Supplement: Supplementary file 1 [file ijerph-17-00690-s001.pdf]

Supplementary Material

Document S1: Assumptions of the General Linear Model

Testing Assumptions for the General Linear Model

Normality, homoscedasticity, multi-collinearity, and linearity assumptions were evaluated before conducting analyses using the General Linear Model.

Histograms revealed that all three outcome measures had slightly negatively skewed distributions and Normal Predicted Probability (P-P) plots revealed slight deviations in the residuals. Thus, the three outcome measures were normalized using Blom's rank based normalization. All three variables displayed normal distributions following transformation, with residuals conforming to the diagonal normality line indicated in the plot. Next, homoscedasticity, linearity, and multicollinearity were evaluated, and all these assumptions were met.

Evaluation of Normality for ATSS Coding Categories

Tests of normality were evaluated for the seven final summary code scores prior to use in multi-level models. This was done on the basis of the values for skewness and kurtosis. The results indicated that none of the seven codes was normally distributed, as slight skewness and kurtosis were present in each of their distributions. Thus, all seven code scores were normalized using Blom's rank based normalization. Normality was again evaluated and all values were found to be within the acceptable range for skewness and kurtosis (below +1.5 and above -1.5).
